# Vitamin D supplementation during intensive care unit stay is associated with improved outcomes in critically Ill patients with sepsis: a cohort study

**DOI:** 10.3389/fcimb.2024.1485554

**Published:** 2025-01-20

**Authors:** Caifeng Li, Ke Zhao, Qian Ren, Lin Chen, Ying Zhang, Guolin Wang, Keliang Xie

**Affiliations:** ^1^ Department of Critical Care Medicine, Tianjin Medical University General Hospital, Tianjin, China; ^2^ Department of General Surgery, Tianjin Medical University General Hospital, Tianjin, China; ^3^ Advertising Center, Tianjin, China; ^4^ Department of Neurosurgery, Tianjin Medical University General Hospital Airport Hospital, Tianjin, China

**Keywords:** critical illness, mortality, supplementation, vitamin D, microbial infection, sepsis

## Abstract

**Background:**

Patients with vitamin D deficiency are susceptible to increased microbial infection and increased risk of mortality. However, whether vitamin D supplementation would improve their prognosis remains uncertain.

**Methods:**

We conducted a retrospective cohort study using data from MIMIC-IV database, a publicly available database containing clinical information on patients admitted to the ICU at Beth Israel Deaconess Medical Center (BIDMC) from 2008 to 2019. Adult patients with sepsis were included in the analysis. The exposure factor was vitamin D supplementation during the ICU stay. The primary outcome was 28-day all-cause mortality. Both propensity score matching (PSM) and stepwise regression analyses were employed to adjust for potential confounders.

**Results:**

A total of 20230 eligible patients were enrolled in the entire unmatched cohort, and 8710 patients were included in the matched cohort. In PSM analysis, the 28-day all-cause mortality rate was 14.04% (250/1780) in the vitamin D group and 22.31% (1546/6930) in the no vitamin D group. Vitamin D supplementation was associated with decreased 28-day all-cause mortality (HR, 0.56; 95% CI, 0.49-0.64; p < 0.001). Subgroup analyses showed consistent benefits regardless of the baseline vitamin D status (deficiency: HR, 0.70; 95% CI, 0.33-1.50; p = 0.36; insufficiency: HR, 0.10; 95% CI, 0.03-0.34; p < 0.001; sufficiency: HR, 0.33; 95% CI, 0.12-0.88; p = 0.03). Additionally, vitamin D supplementation was associated with decreased ICU mortality (OR, 0.37; 95% CI, 0.29-0.48; p < 0.001) and reduced in-hospital mortality (OR, 0.57; 95% CI, 0.48-0.68; p < 0.001). Sensitivity analysis using the unmatched cohort confirmed these findings (HR, 0.57; 95% CI, 0.43-0.76; p < 0.001).

**Conclusions:**

Vitamin D supplementation may reduce mortality in critically ill patients with sepsis. However, further high-quality prospective studies are still needed to validate these findings.

## Background

Sepsis, defined as a syndrome of life-threatening organ failure due to a dysregulated host immune response to infection (Singer et al., 2016), is the major cause of morbidity and mortality in critically ill patients worldwide, with an estimated 31.5 million cases of sepsis, 19.4 million cases of severe sepsis, and up to 5.3 million deaths annually (Fleischmann et al., 2016). The 2021 Surviving Sepsis Campaign Guidelines (Evans et al., 2021) advocated the timely implementation of standardized intervention bundles to alleviate the burden of sepsis, which include early patient identification, effective infection source control, appropriate antimicrobial therapy and sufficient organ support. Although the essence of sepsis is a systemic uncontrolled inflammatory response caused by infection, there are no adjunctive treatments that directly target immune dysregulation other than systemic corticosteroid use in certain clinical circumstances (de Gans and van de Beek, 2002; Torres et al., 2015; Annane et al., 2018; Yanase et al., 2020; Cascarano et al., 2021; Cutuli et al., 2021b; Cutuli et al., 2021a; De Rosa et al., 2021; Horby et al., 2021; Dequin et al., 2023), which results in systemic immunosuppression.

In this context, an increasing amount of academic literature demonstrated the potential role of vitamin D in restoring immune system homeostasis and ameliorating the adverse consequences of a dysregulated immune response (Colotta et al., 2017; Cutuli et al., 2022; Cao et al., 2023). Vitamin D is a fat-soluble secosteroid hormone whose metabolism depends on sunlight exposure, dietary intake, the liver and kidney functions. Insufficient sunlight exposure, inadequate dietary intake, and acute or chronic liver or kidney disease can disrupt its metabolism (Cutuli et al., 2022), leading to vitamin D insufficiency or deficiency. Epidemiologic studies have demonstrated that vitamin D deficiency is common in the community (Cashman et al., 2016; Herrick et al., 2019) and is prevalent in critically ill patients, with an estimated incidence of 70% among those admitted to the intensive care unit (ICU) (Amrein et al., 2018). Vitamin D deficiency is associated with severe infections, sepsis, and poor clinical outcomes (de Haan et al., 2014; De Pascale et al., 2016; McNally et al., 2017; Parekh et al., 2017). Previous studies have shown the feasibility, safety and effectiveness of vitamin D supplementation strategies in improving vitamin D levels in critical care settings (Amrein et al., 2011; Quraishi et al., 2015). Early vitamin D supplementation seems to be theoretically well-founded, particularly in the vulnerable sepsis subgroup (Singer et al., 2016; Sinha et al., 2021). However, conflicting findings cast doubt on its impact on patient prognosis (Amrein et al., 2014; Leaf et al., 2014; Ginde et al., 2019; Murai et al., 2021). Previous randomized controlled trials (RCTs) and meta-analyses have suggested that vitamin D could potentially decrease the incidence of septic shock (Wang et al., 2020), shorten the duration of mechanical ventilation (MV) and length of ICU stay (Singh et al., 2022), and reduce the mortality rate in critically ill patients (Menger et al., 2022). However, unsatisfactory outcomes have been reported by other researchers, with increased mortality rates in the vitamin D group, with higher mortality rates observed in the vitamin D group (Ginde et al., 2019; Bhattacharyya et al., 2021). Currently, whether vitamin D supplementation would be beneficial for critically ill patients is still controversial.

Differences in the findings of recent studies may be due to the heterogeneity of critically ill patients and the limited sample size of clinical trial cohorts. Large-scale RCTs or real-world studies are essential to draw robust conclusions. Therefore, we conducted a retrospective cohort study using the Medical Information Mart in Intensive Care-IV (MIMIC-IV) database to investigate the association of vitamin D supplementation with mortality and the need for organ support in critically ill patients with sepsis.

## Materials and methods

### Study design and data source

This retrospective propensity score matched cohort study was based on the MIMIC-IV (Johnson et al., 2023), a publicly available database containing de-identified health information of patients admitted to the intensive care units of the Beth Israel Deaconess Medical Center (BIDMC) in Boston, Massachusetts, between 2008 and 2019. The database contained a variety of clinical data for each patient, such as demographics, vital signs, laboratory results, hospital/ICU admission and discharge time, ICU transfer date, prescriptions, nursing records, and other information. Informed consent was waived by the Institutional Review Board at BIDMC due to the de-identified nature of this database. The author, Li, passed the Examination of Protection of Human Research Participants and was granted access to the database for data extraction (record ID: 33047414). The study was reported following the Strengthening the Reporting of Observational Studies in Epidemiology (STROBE) statement (von Elm et al., 2007).

### Study population

All consecutive adult patients of both sexes were screened according to the following inclusion criteria: (1) Diagnosed with sepsis (Sepsis-3)(1) upon hospital admission. (2) Aged 18 years or older. (3) For patients with multiple ICU stays, only data related to the first ICU stay were included. Patients with an ICU stay of less than 24 hours were also excluded.

### Exposure and outcomes

Medication exposure was identified using the prescription table. Vitamin D exposure was defined simply as vitamin D supplementation during the ICU stay, with no other restrictions. The route of vitamin D administration could vary, either intravenous administration (IV), oral administration (PO) or nasogastric feeding (NG). The primary outcome was 28-day all-cause mortality. The secondary outcomes included ICU mortality, in-hospital mortality, length of ICU stay, length of hospital stay, duration of MV and duration of continuous renal replacement therapy (CRRT). According to vitamin D exposure during ICU stay, all eligible patients were categorized into either the vitamin D group or the no vitamin D group to assess the effect of vitamin D supplementation on clinical outcomes.

### Data extraction

All data were extracted using Structured Query Language (SQL). The SQL script codes for data extraction were available on GitHub (https://github.com/MIT-LCP/mimic-iv). The following data were collected: demographics, including age, gender, race and body mass index (BMI); vital signs, such as respiratory rate (RR), heart rate, temperature and mean blood pressure (MBP); comorbidities, including congestive heart failure, cerebrovascular disease, chronic pulmonary disease, diabetes, renal disease, cancer, severe liver disease; severity scores, including acute physiology score III (APS III), charlson comorbidity index (CCI), logistic organ dysfunction system (LODS), oxford acute severity of illness score (OASIS), sequential organ failure assessment (SOFA), glascow coma scale (GCS); laboratory tests, including 25-hydroxyvitamin D, hemoglobin, platelets, white blood cell (WBC), blood urea nitrogen (BUN), creatinine, alanine aminotransferase (ALT), aspartate aminotransferase (AST), total bilirubin, glucose, potential of hydrogen (pH), partial pressure of oxygen (pO2), partial pressure of carbon dioxide (pCO2), partial pressure of arterial oxygen to fraction of inspired oxygen ratio (PaO2/FiO2 ratio), base excess, lactate, calcium, sodium, potassium, chloride, anion gap, international normalized ratio (INR); clinical measures, including antibiotic lag, first-day vasopressor, duration of MV, duration of CRRT. Comorbidities were assessed on admission. Initial vital signs and clinical indices obtained within the first 24 hours after ICU admission were used as baseline characteristics. Variables with missing values of more than 50% were excluded, while the rest variables were included in the study. The missing rate for each variable was presented in **Supplementary Table S1** and **Supplementary Figure S1**. Missing values for each variable were imputed using the missForest method (Sterne et al., 2009).

### Statistical analysis

No prior statistical power calculation was performed, as the sample size for this retrospective study was determined only by the number of eligible patients in the MIMIC-IV database. Continuous variables were expressed as either mean (standard deviation [SD]) or median (interquartile range [IQR]) and analyzed using either the Students’ t-test or the Mann-Whitney’s U-test, depending on their distribution. Categorical variables were presented as numbers and percentages and examined using either the chi-square test or Fisher’s exact test. For the primary outcome, the Cox proportional hazards model was employed to calculate the hazard ratio (HR) and 95% confidence interval (CI). The Kaplan-Meier method and the log-rank test were used to estimate and compare the cumulative incidence of 28-day all-cause mortality. For dichotomous secondary outcomes, the logistic regression model was applied to calculate the odds ratio (OR) and 95% CI. For continuous secondary outcomes, the Hodgese-Lehmann method was utilized to calculate the median difference (MD) and 95% CI. Potential multicollinearity between variables was evaluated using the variance inflation factor (VIF), with VIF below 5 indicating the absence of multicollinearity (**Supplementary Table S2**, **Supplementary Figure S2**; **Supplementary Table S3**, **Supplementary Figure S3**). For all analyses, a two-tailed p < 0.05 was deemed statistically significant. All statistical analyses and visualizations were conducted using R software (version 4.2.3; R Foundation for Statistical Computing, Vienna, Austria).

### Propensity score matching

To reduce the impact of potential confounding factors and selection bias inherent in observational studies, PSM was performed following the methodological guidelines proposed by Lonjon and colleagues (Lonjon et al., 2017). According to a consensus statement (Cecconi et al., 2018), the following variables were included in the propensity score model: age, gender, race, BMI, diabetes, renal disease, severe liver disease, OASIS, GCS, MBP, heart rate, first care unit, hemoglobin, BUN, creatinine, glucose, pO2, PaO2/FiO2 ratio, base excess, calcium, chloride, anion gap, INR, antibiotic lag and first-day vasopressor. The propensity score, the predicted probability of receiving vitamin D supplementation, was calculated by using baseline covariates in a logistic regression model. Patients were matched using the 1:4 nearest neighbor method with no replacement and a caliper width of 0.05. After PSM, a matched cohort of patients with similar baseline characteristics was assembled. Covariate balance between groups was assessed using standardized mean differences (SMD) before and after matching, with SMD < 0.1 indicating insignificant differences (Austin, 2009). Stepwise Cox regression analyses were also employed to screen independent prognostic factors and adjust for confounding factors. In the matched cohort dataset, variables with p-value < 0.1 in the univariable analysis were included as candidate variables in the multivariable analysis by stepwise selection. The independent variables included in the final model were age, race, BMI, APS-III, Charlson Comorbidity Index, LODS, OASIS, SOFA, GCS, MBP, respiratory rate, heart rate, temperature, hemoglobin, WBC, BUN, creatinine, AST, total bilirubin, glucose, pH, pO2, PaO2/FiO2 ratio, base excess, lactate, potassium, chloride, anion gap, INR, antibiotic lag, first-day vasopressor, vitamin D supplementation (**Supplementary Table S4**).

### Subgroup analyses

To determine the effect of different variables on 28-day all-cause mortality in patients with sepsis, we conducted a subgroup analysis within the matched cohort according to age (>60 vs. <=60 years), gender (female vs. male), race (white, black, unknown, other), BMI (obesity, overweight, normal, underweight), Charlson Comorbidity Index (<6 vs. >=6) and serum 25-hydroxyvitamin D (deficiency with 25-hydroxyvitamin D levels < 20 ng/mL, insufficiency with 25-hydroxyvitamin D levels between 20 and 29 ng/mL vs. sufficiency with 25-hydroxyvitamin D levels >= 30 ng/mL).

### Sensitivity analysis

To verify the reliability of the findings from the matched cohort, we performed a sensitivity analysis in the unmatched cohort. Stepwise Cox regression analyses were employed to identify independent prognostic factors and adjust for potential confounders. Variables with p-value < 0.1 in the univariable analysis were integrated into the multivariable analysis to adjust for potential confounding factors. The independent variables included in the final model were: age, gender, race, BMI, APS-III, Charlson Comorbidity Index, LODS, OASIS, SOFA, GCS, MBP, respiratory rate, heart rate, temperature, hemoglobin, WBC, BUN, creatinine, ALT, AST, total bilirubin, pH, pO2, pCO2, PaO2/FiO2 ratio, base excess, lactate, Calcium, Sodium, potassium, chloride, anion gap, INR, antibiotic lag, first-day vasopressor, vitamin D supplementation (**Supplementary Table S5**).

## Result

### Patient selection

**Figure 1:**
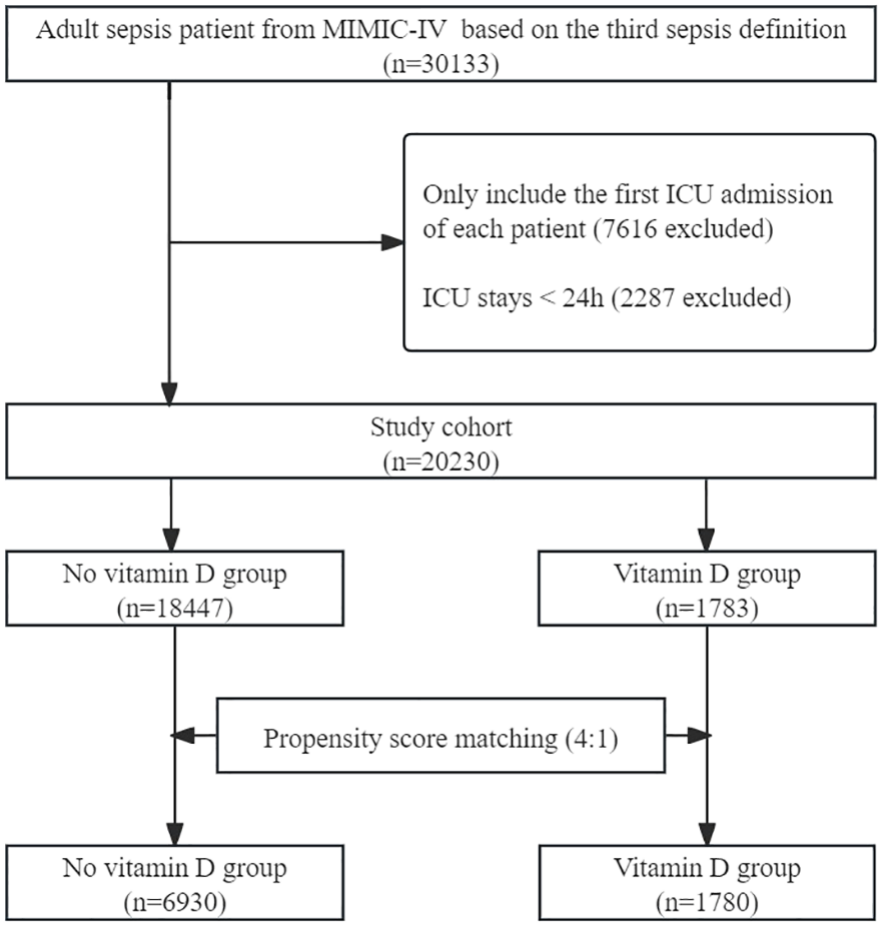
Flow chart of patient selection. MIMIC-IV, Medical Information Mart in Intensive Care-IV.

The process of patient selection is depicted in **Figure 1**. A total of 30133 adult patients with sepsis were identified during the study period. Following the removal of ineligible records, 20230 patients were included in the entire unmatched cohort, among which 1783 (8.81%) received vitamin D supplementation during their ICU stay. After matching, 8710 patients were included in the matched cohort, with 6930 in the no vitamin D group and 1780 in the vitamin D group.

### Cohort characteristics

**Figure 2:**
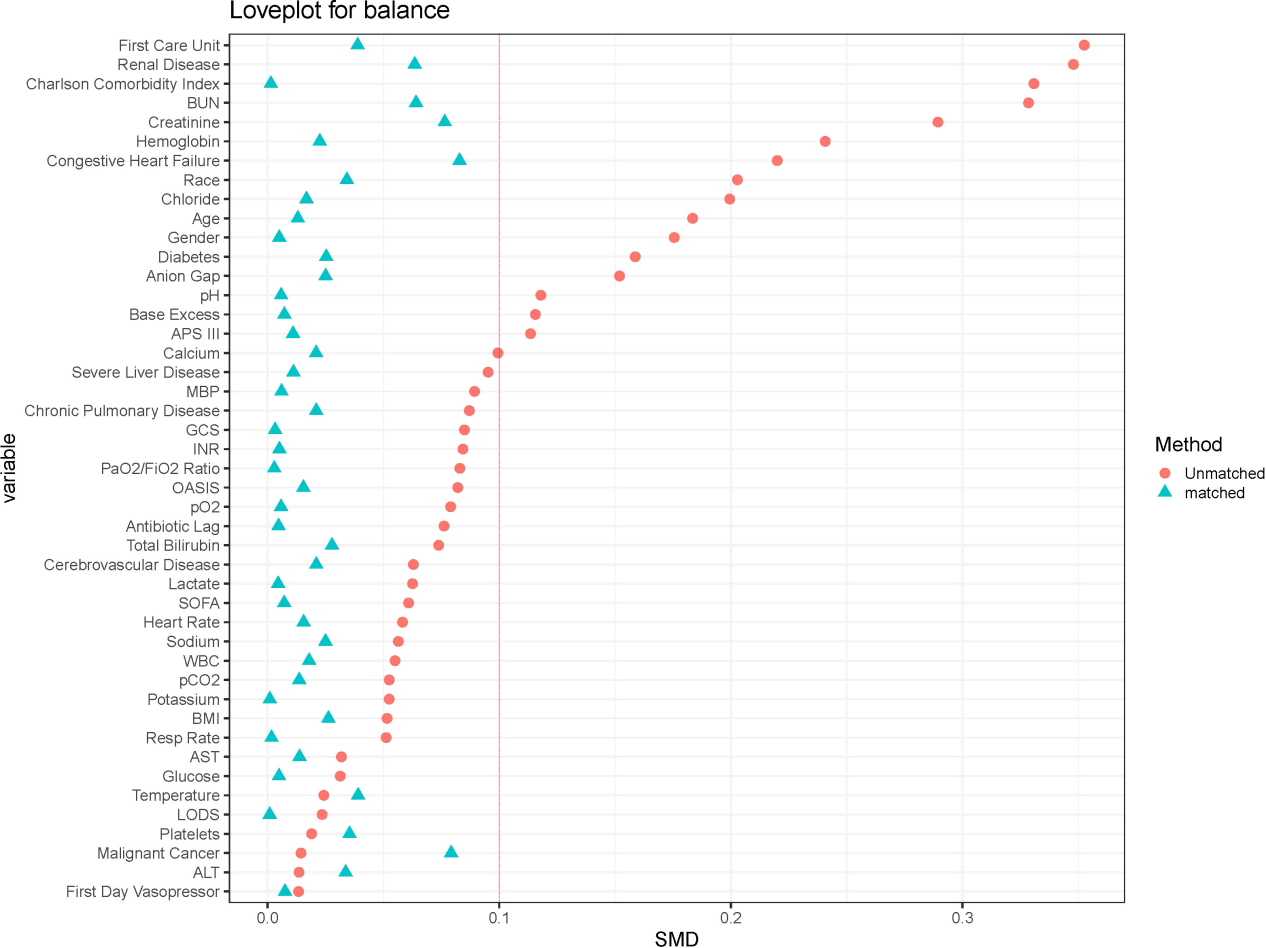
The loveplot showed SMD across covariates before and after propensity score matching.

**Figure 3:**
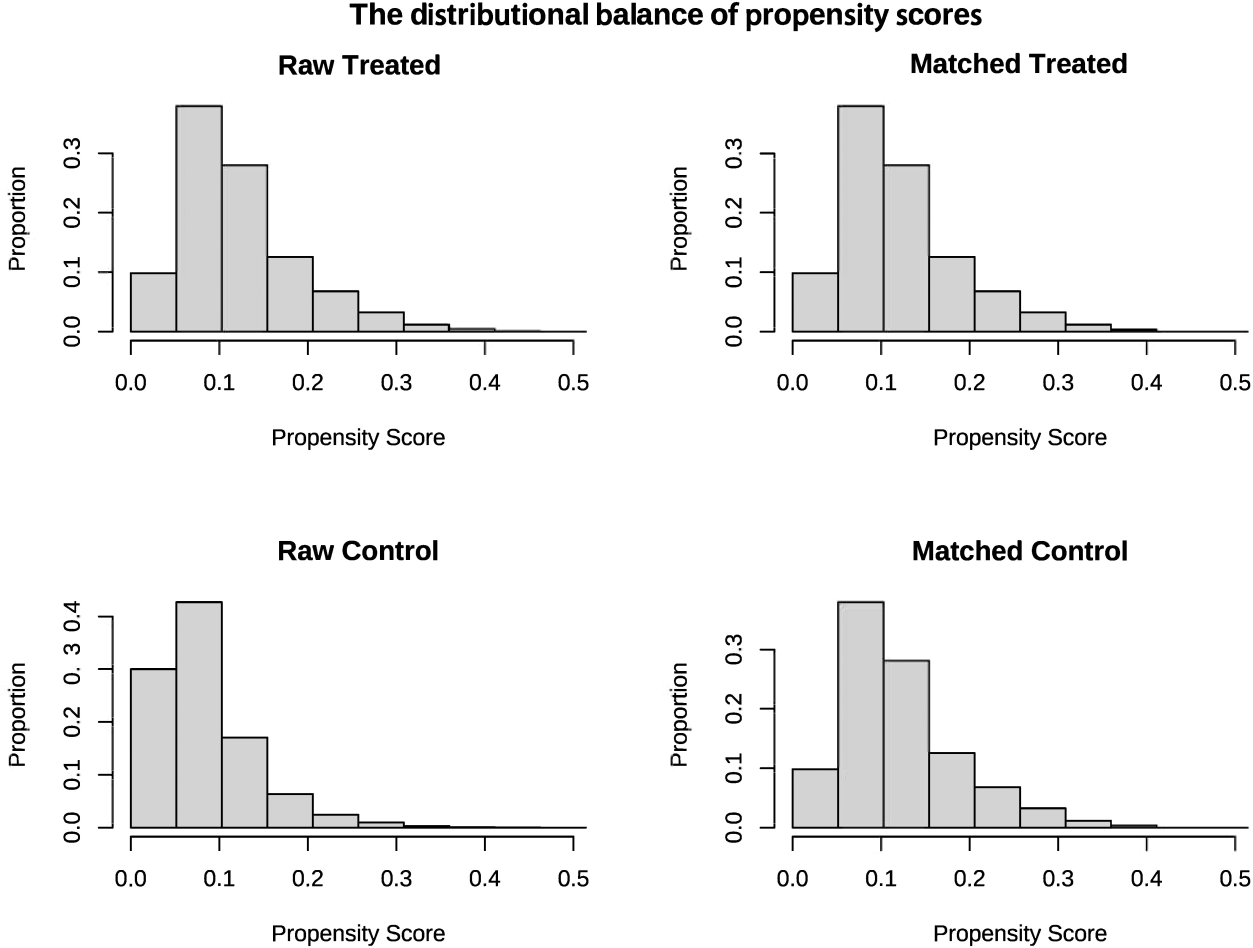
The distributional balance of propensity scores before and after propensity score matching in the two groups.

The baseline characteristics of the unmatched and matched cohort are shown in **Table 1**. In the unmatched cohort, patients in the vitamin D group tended to be older and more likely to be female, exhibited higher APS-III and Charlson Comorbidity Index, lower serum 25-hydroxyvitamin D levels and longer antibiotic lag. In the matched cohort, propensity score-matched variables were well balanced, with an SMD < 0.10, indicating that no imbalances remained for variables included in the PSM (**Figure 2**). The distributional balance of propensity scores for both groups before and after PSM is presented in **Figure 3**.

**Table 1:** Baseline characteristics before and after propensity score matching.

Variable	Before propensity score matching					After propensity score matching				
	Overall	No vitamin D	Vitamin D	p	SMD	Overall	No vitamin D	Vitamin D	p	SMD
n	20230	18447	1783			8710	6930	1780		
Age (yr), n (%)										
<=60	6359 (31.4)	5932 (32.2)	427 (23.9)	<0.001	0.183	2046 (23.5)	1620 (23.4)	426 (23.9)	0.64	0.013
>60	13871 (68.6)	12515 (67.8)	1356 (76.1)			6664 (76.5)	5310 (76.6)	1354 (76.1)		
Gender, n (%)										
Female	8458 (41.8)	7571 (41.0)	887 (49.7)	<0.001	0.176	4313 (49.5)	3428 (49.5)	885 (49.7)	0.87	0.005
Male	11772 (58.2)	10876 (59.0)	896 (50.3)			4397 (50.5)	3502 (50.5)	895 (50.3)		
Race, n (%)										
Black	1576 (7.8)	1379 (7.5)	197 (11.0)	<0.001	0.203	904 (10.4)	708 (10.2)	196 (11.0)	0.64	0.034
White	13564 (67.0)	12318 (66.8)	1246 (69.9)			6163 (70.8)	4918 (71.0)	1245 (69.9)		
Other	2074 (10.3)	1910 (10.4)	164 (9.2)			820 (9.4)	657 (9.5)	163 (9.2)		
Unknown	3016 (14.9)	2840 (15.4)	176 (9.9)			823 (9.4)	647 (9.3)	176 (9.9)		
BMI (kg/m^2^), median [IQR]	28 [24, 32]	28 [24, 32]	27 [23, 32]	0.02	0.052	28 [25, 31]	28 [25, 31]	28 [25, 31]	0.44	0.026
Comorbidities, n (%)										
Congestive Heart Failure	5755 (28.4)	5081 (27.5)	674 (37.8)	<0.001	0.22	3018 (34.6)	2345 (33.8)	673 (37.8)	0.002	0.083
Cerebrovascular Disease	2964 (14.7)	2738 (14.8)	226 (12.7)	0.02	0.063	1155 (13.3)	929 (13.4)	226 (12.7)	0.46	0.021
Chronic Pulmonary Disease	5229 (25.8)	4705 (25.5)	524 (29.4)	<0.001	0.087	2498 (28.7)	1974 (28.5)	524 (29.4)	0.45	0.021
Diabetes	6059 (30.0)	5404 (29.3)	655 (36.7)	<0.001	0.159	3111 (35.7)	2458 (35.5)	653 (36.7)	0.35	0.025
Renal Disease	4223 (20.9)	3603 (19.5)	620 (34.8)	<0.001	0.348	2812 (32.3)	2195 (31.7)	617 (34.7)	0.02	0.064
Malignant Cancer	2700 (13.3)	2454 (13.3)	246 (13.8)	0.58	0.014	1401 (16.1)	1155 (16.7)	246 (13.8)	0.004	0.079
Severe Liver Disease	1407 (7.0)	1241 (6.7)	166 (9.3)	<0.001	0.095	835 (9.6)	669 (9.7)	166 (9.3)	0.71	0.011
Severity score, median [IQR]										
APS III	45 [34, 61]	45 [33, 61]	49 [37, 62]	<0.001	0.114	48 [37, 62]	48 [36, 62]	49 [37, 62]	0.27	0.011
CCI	5 [3, 7]	5 [3, 7]	6 [4, 8]	<0.001	0.331	6 [4, 8]	6 [4, 8]	6 [4, 8]	0.91	0.001
LODS	5 [3, 8]	5 [3, 8]	5 [3, 8]	0.06	0.024	5 [3, 8]	5 [3, 8]	5 [3, 8]	0.67	0.001
OASIS	34 [28, 41]	35 [28, 41]	34 [28, 40]	0.001	0.082	34 [28, 40]	34 [28, 40]	34 [28, 40]	0.54	0.015
SOFA	5 [3, 8]	5 [3, 8]	5 [4, 8]	0.001	0.061	5 [3, 8]	5 [3, 8]	5 [4, 8]	0.38	0.007
GCS	15 [13, 15]	15 [13, 15]	15 [13, 15]	0.49	0.085	15 [13, 15]	15 [13, 15]	15 [13, 15]	0.98	0.003
Vital signs, median [IQR]										
MBP (mmHg)	75.40 [70.01, 82.06]	75.47 [70.10, 82.13]	74.52 [69.13, 81.36]	<0.001	0.089	74.56 [69.00, 81.40]	74.56 [68.96, 81.41]	74.52 [69.14, 81.36]	0.88	0.006
Respiratory Rate (bpm)	18.91 [16.69, 21.90]	18.87 [16.67, 21.87]	19.33 [16.90, 22.13]	0.003	0.051	19.23 [16.89, 22.17]	19.20 [16.88, 22.19]	19.32 [16.90, 22.11]	0.79	0.002
Heart Rate (bpm)	85.21 [75.64, 97.03]	85.27 [75.75, 97.08]	84.30 [74.57, 96.48]	0.02	0.058	84.78 [74.67, 96.68]	84.93 [74.69, 96.68]	84.31 [74.58, 96.50]	0.45	0.016
Temperature (°C)	36.86 [36.60, 37.22]	36.87 [36.60, 37.22]	36.83 [36.61, 37.13]	0.01	0.024	36.82 [36.58, 37.14]	36.82 [36.57, 37.14]	36.82 [36.61, 37.12]	0.56	0.039
First Care Unit, n (%)										
CVICU	4543 (22.5)	4313 (23.4)	230 (12.9)	<0.001	0.353	1074 (12.3)	844 (12.2)	230 (12.9)	0.71	0.039
MICU	4330 (21.4)	3798 (20.6)	532 (29.8)			2552 (29.3)	2022 (29.2)	530 (29.8)		
MICU/SICU	3697 (18.3)	3269 (17.7)	428 (24.0)			2081 (23.9)	1653 (23.9)	428 (24.0)		
SICU	2790 (13.8)	2574 (14.0)	216 (12.1)			1122 (12.9)	907 (13.1)	215 (12.1)		
Other	4870 (24.1)	4493 (24.4)	377 (21.1)			1881 (21.6)	1504 (21.7)	377 (21.2)		
Laboratory tests, median [IQR]										
Hemoglobin (g/dL)	9.7 [8.3, 11.3]	9.8 [8.4, 11.3]	9.3 [8.0, 10.8]	<0.001	0.241	9.3 [8.0, 10.8]	9.3 [8.0, 10.8]	9.3 [8.0, 10.8]	0.27	0.023
Platelets (10^9^/L)	158.0 [109.0, 221.0]	158.0 [110.0, 221.0]	158.0 [106.0, 229.0]	0.71	0.019	162.0 [107.0, 233.0]	163.0 [107.0, 234.0]	158.50 [106.0, 229.0]	0.37	0.035
WBC (10^9^/L)	14.0 [10.1, 18.9]	14.1 [10.2, 19.0]	12.8 [8.9, 18.0]	<0.001	0.055	13.3 [9.4, 18.4]	13.4 [9.5, 18.5]	12.8 [8.9, 18.0]	0.002	0.018
BUN (mg/dL)	22.0 [15.0, 37.0]	22.0 [15.0, 36.0]	29.0 [18.0, 50.0]	<0.001	0.328	28.0 [18.0, 47.0]	28.0 [18.0, 46.0]	29.0 [18.0, 50.0]	0.09	0.064
Creatinine (mg/dL)	1.1 [0.8, 1.8]	1.1 [0.8, 1.7]	1.4 [0.9, 2.5]	<0.001	0.289	1.3 [0.9, 2.2]	1.3 [0.9, 2.2]	1.4 [0.9, 2.5]	0.052	0.076
ALT (U/L)	31.0 [18.0, 79.0]	32.0 [18.0, 80.0]	26.0 [15.0, 65.0]	<0.001	0.014	60.9 [22.0, 115.0]	61.8 [23.0, 116.0]	55.0 [20.0, 111.3]	0.003	0.034
AST (U/L)	48.0 [27.0, 128.0]	49.0 [27.0, 131.0]	41.0 [24.0, 100.0]	<0.001	0.032	85.0 [34.0, 157.5]	86.3 [35.0, 159.5]	79.0 [31.0, 151.0]	0.001	0.014
Total Bilirubin (mg/dL)	0.8 [0.4, 1.8]	0.8 [0.5, 1.8]	0.7 [0.4, 1.9]	0.01	0.074	1.2 [0.6, 2.2]	1.2 [0.6, 2.3]	1.2 [0.5, 2.2]	0.09	0.028
Glucose (mg/dL)	131.6 [115.0, 159.0]	131.5 [115.2, 158.2]	133.0 [112.0, 165.0]	0.50	0.031	133.0 [112.7, 165.0]	133.0 [113.0, 165.2]	133.0 [112.0, 164.9]	0.77	0.005
pH	7.32 [7.26, 7.38]	7.32 [7.26, 7.38]	7.33 [7.27, 7.39]	0.001	0.118	7.34 [7.31, 7.38]	7.34 [7.31, 7.38]	7.34 [7.31, 7.37]	0.41	0.006
pO2 (mmHg)	92.0 [73.0, 123.0]	92.0 [73.0, 124.0]	86.0 [70.0, 116.0]	<0.001	0.079	98.0 [80.0, 123.4]	98.0 [80.0, 124.0]	98.1 [80.0, 122.4]	0.97	0.006
pCO2 (mmHg)	46.0 [40.0, 52.0]	46.0 [40.0, 52.0]	44.0 [38.0, 51.0]	<0.001	0.053	43.0 [39.3, 47.2]	43.0 [39.3, 47.6]	43.0 [39.3, 47.0]	0.54	0.014
PaO2/FiO2 Ratio	196.7 [128.0, 281.1]	197.5 [128.0, 282.5]	185.7 [126.3, 265.0]	0.04	0.083	219.0 [169.5, 282.1]	219.3 [168.3, 282.5]	218.6 [171.4, 280.0]	0.92	0.003
Base Excess (mmol/L)	-3.0 [-6.0, 0.0]	-3.0 [-6.0, 0.0]	-2.0 [-6.0, 0.0]	<0.001	0.116	-2.0 [-4.4, 0.0]	-2.0 [-4.4, 0.0]	-2.0 [-4.4, 0.0]	0.72	0.007
Lactate (mmol/L)	2.3 [1.5, 3.5]	2.3 [1.5, 3.6]	2.0 [1.4, 3.3]	<0.001	0.063	2.1 [1.6, 2.7]	2.1 [1.6, 2.8]	2.0 [1.6, 2.7]	0.35	0.005
Calcium (mg/dL)	8.0 [7.5, 8.5]	8.0 [7.5, 8.5]	8.1 [7.5, 8.6]	<0.001	0.099	8.1 [7.5, 8.5]	8.1 [7.5, 8.5]	8.1 [7.6, 8.5]	0.09	0.021
Sodium (mmol/L)	137.0 [134.0, 140.0]	137.0 [134.0, 140.0]	137.0 [134.0, 140.0]	0.08	0.056	137.0 [134.0, 140.0]	137.0 [134.0, 140.0]	137.0 [134.0, 140.0]	0.14	0.025
Potassium (mmol/L)	4.5 [4.1, 5.0]	4.5 [4.1, 4.9]	4.5 [4.1, 5.1]	0.25	0.052	4.5 [4.1, 5.0]	4.5 [4.1, 5.0]	4.5 [4.1, 5.1]	0.88	0.001
Chloride (mmol/L)	103.0 [99.0, 106.0]	103.0 [99.0, 106.0]	102.0 [97.0, 106.0]	<0.001	0.199	102.0 [97.0, 106.0]	102.0 [97.0, 106.0]	102.0 [97.0, 106.0]	0.45	0.017
Anion Gap (mmol/L)	16.0 [13.0, 19.0]	16.0 [13.0, 19.0]	16.0 [14.0, 20.0]	<0.001	0.152	16.0 [14.0, 20.0]	16.0 [14.0, 20.0]	16.0 [14.0, 20.0]	0.34	0.025
INR	1.3 [1.2, 1.6]	1.3 [1.2, 1.6]	1.4 [1.2, 1.7]	0.002	0.084	1.4 [1.2, 1.7]	1.4 [1.2, 1.7]	1.4 [1.2, 1.8]	0.26	0.005
25(OH)D (ng/mL)	21.0 [13.0, 31.0]	24.0 [16.0, 34.0]	17.0 [11.0, 26.0]	<0.001	0.421	20.0 [13.0, 31.0]	26.0 [17.0, 35.0]	17.0 [11.0, 26.0]	<0.001	0.517
Treatment										
Antibiotic Lag (hrs), median [IQR]	7.10 [1.55, 18.00]	7.00 [1.38, 18.00]	8.60 [3.98, 18.72]	<0.001	0.076	8.00 [3.25, 19.15]	7.94 [3.02, 19.23]	8.61 [3.94, 18.78]	0.03	0.005
First Day Vasopressor, n (%)	5944 (29.4)	5430 (29.4)	514 (28.8)	0.61	0.013	2534 (29.1)	2021 (29.2)	513 (28.8)	0.80	0.008

SMD, Standardized Mean Difference; IQR, Interquartile Range; APS III, Acute Physiology Score III; CCI, Charlson Comorbidity Index; LODS, Logistic Organ Dysfunction System; OASIS, Oxford Acute Severity of Illness Score; SOFA, Sequential Organ Failure Assessment Score; GCS, Glasgow Coma Scale; MBP, Mean Blood Pressure; WBC, White Blood Cell; BUN, Blood Urea Nitrogen; ALT, Alanine Aminotransferase; AST, Aspartate Aminotransferase; Ph, Potential of Hydrogen; pO2, partial pressure of Oxygen; pCO2, partial ressure of Carbon Dioxide; INR, International Normalized Ratio; CVICU, Cardiac Vascular Intensive Care Unit; MICU, Medical Intensive Care Unit; MICU/SICU, Medical/Surgical Intensive Care Unit; SICU, Surgical Intensive Care Unit; PaO2/FiO2 Ratio, partial pressure of arterial oxygen to fraction of inspired oxygen ratio; 25(OH)D, 25-hydroxyvitamin D.

### Relationship between serum 25-hydroxyvitamin D and 28-day mortality in sepsis

The relationship between serum 25-hydroxyvitamin D and 28-day mortality in septic patients was examined through the RCS curves in the matched cohort (**Supplementary Figure S4**). The RCS curve showed nearly linear relationship. between 28-day mortality and serum 25-hydroxyvitamin D on a continuous scale, suggesting that excessively low serum 25-hydroxyvitamin D increase the risk of mortality in septic patients.

### Vitamin D regimen

The prevalence of vitamin D deficiency in the unmatched cohort was 1.12% (226/20230), with 7.35% (131/1783) in the vitamin D group and 0.51% (95/18447) in the no vitamin D group (p < 0.001). Vitamin D insufficiency was observed in 0.63% (127/20230) of the unmatched cohort, including 3.20% (57/1783) in the vitamin D group and 0.38% (70/18447) in the no vitamin D group (p < 0.001). The prevalence of vitamin D sufficiency was 0.69% (139/20230) in the unmatched cohort, comprising 2.24% (40/1783) in the vitamin D group and 0.54% (99/18447) in the no vitamin D group (p < 0.001).

The prevalence of vitamin D deficiency in the matched cohort was 2.07% (180/8710), with 7.36% (131/1780) in the vitamin D group and 0.71% (49/6930) in the no vitamin D group (p < 0.001). Vitamin D insufficiency was observed in 1.09% (95/8710) of the matched cohort, including 3.20% (57/1780) in the vitamin D group and 0.55% (38/6930) in the no vitamin D group (p < 0.001). The prevalence of vitamin D sufficiency was 1.18% (103/8710) in the matched cohort, comprising 2.25% (40/1780) in the vitamin D group and 0.91% (63/6930) in the no vitamin D group (p < 0.001).

Clinical indications for initiating and discontinuing vitamin D therapy were unavailable in the database.

### Primary outcome

#### 28-day all-cause mortality

**Figure 4:**
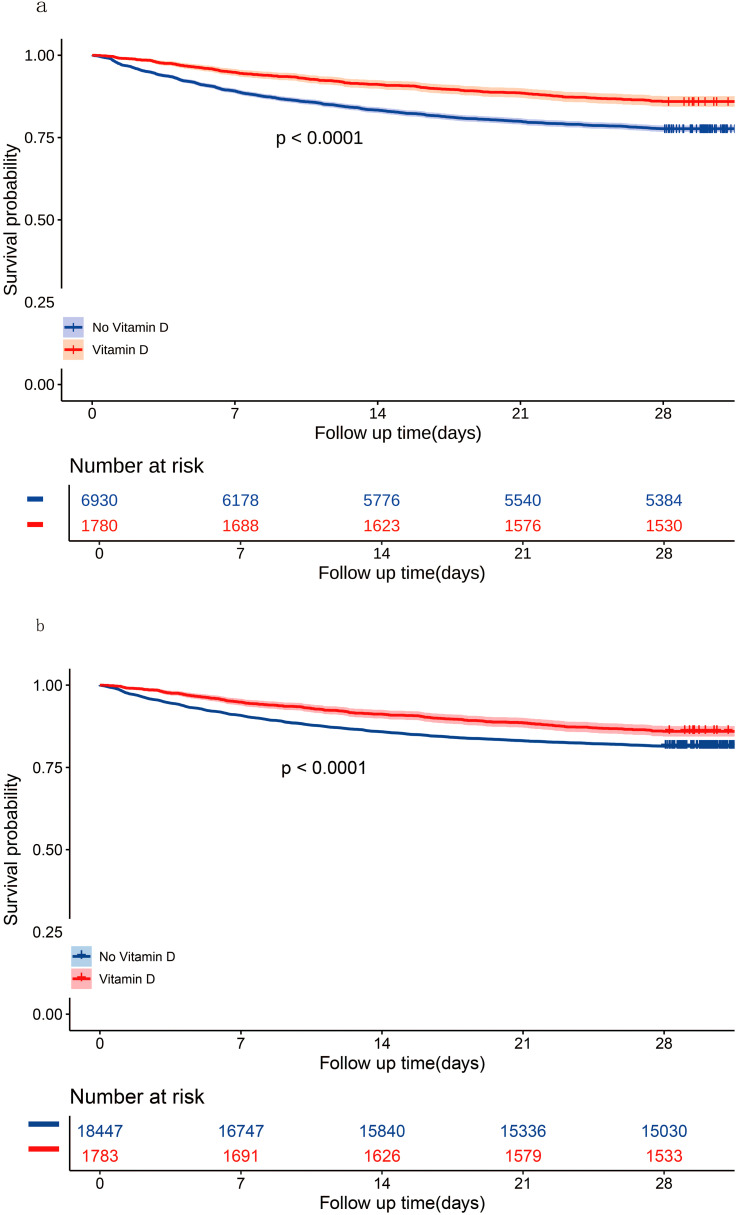
Kaplan-Meier curves for 28-day all-cause mortality according to vitamin D supplementation in the matched cohort **(A)** and the unmatched cohort **(B)**.

In the matched cohort, the 28-day all-cause mortality rate was 14.04% (250/1780) in the vitamin D group and 22.31% (1546/6930) in the no vitamin D group (p < 0.001). **Figure 4A** displays the Kaplan-Meier curve for 28-day all-cause mortality stratified by vitamin D supplementation in the matched cohort. Cox regression analysis indicated that vitamin D supplementation was associated with reduced 28-day all-cause mortality in both univariable analysis (HR, 0.59; 95% CI, 0.52-0.68; p < 0.001) and multivariable analysis (HR, 0.56; 95% CI, 0.49-0.64; p < 0.001) in the matched cohort.

#### Subgroup analyses

**Figure 5:**
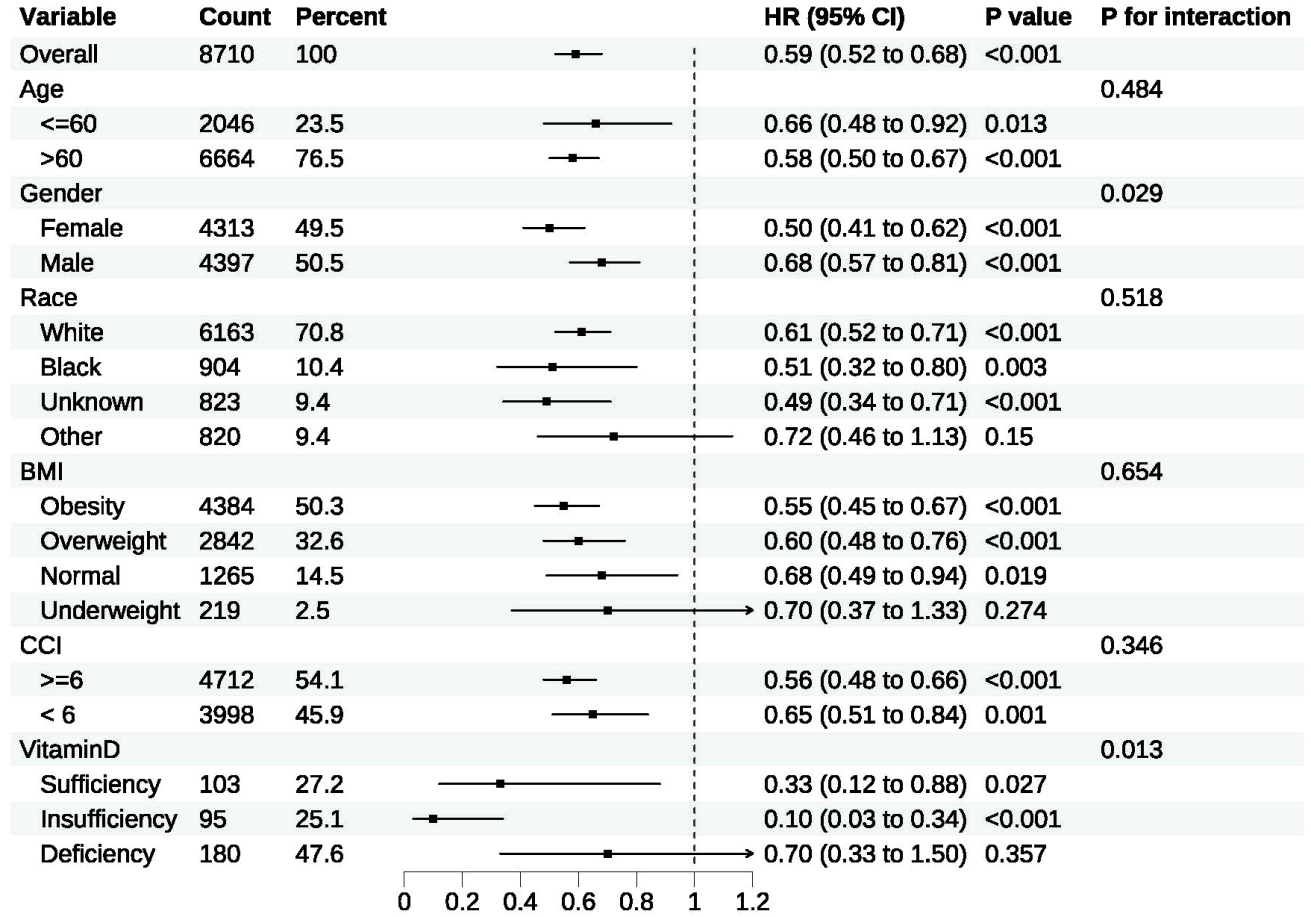
Subgroup analyses for 28-day all-cause mortality in the matched cohort.

The results of subgroup analyses for 28-day all-cause mortality in the matched cohort are demonstrated in **Figure 5**. Vitamin D supplementation was associated with improved clinical outcomes in both the vitamin D insufficiency subgroup (HR, 0.10; 95% CI, 0.03-0.34; p < 0.001) and the vitamin D sufficiency subgroup (HR, 0.33; 95% CI, 0.12-0.88; p = 0.03), whereas vitamin D supplementation was not associated with improved clinical outcomes in the vitamin D deficiency subgroup (HR, 0.70; 95% CI, 0.33-1.50; p = 0.36). Furthermore, except for the underweight subgroup based on BMI and the ‘other’ subgroup based on race, the upper limits of the 95% CIs for all remaining subgroups were less than 1.00, indicating a reduction in 28-day all-cause mortality following vitamin D supplementation irrespective of baseline characteristics. However, given the limited sample size of the underweight subgroup based on BMI, the ‘other’ subgroup based on race and the vitamin D deficiency subgroup based on serum 25-hydroxyvitamin, the findings might be attributable to chance and should be interpreted cautiously.

#### Sensitivity analyses

In the unmatched cohort, the 28-day all-cause mortality rate was 14.02% (250/1783) in the vitamin D group and 18.52% (3417/18447) in the no vitamin D group (p < 0.001). **Figure 4B** shows the Kaplan-Meier curve for 28-day all-cause mortality stratified by vitamin D supplementation in the unmatched cohort. Cox regression analysis indicated that vitamin D supplementation was associated with reduced 28-day all-cause mortality in both univariable analysis (HR, 0.73; 95% CI, 0.64-0.83; p < 0.001) and multivariable analysis (HR, 0.57; 95% CI, 0.42-0.76; p < 0.001) in the unmatched cohort.

### Secondary outcomes

#### ICU mortality and in-hospital mortality

The ICU mortality rate was 5.73% (102/1780) in the vitamin D group and 12.14% (841/6930) in the no vitamin D group (p < 0.001). Logistic regression showed that vitamin D supplementation was associated with a lower ICU mortality rate in both univariable analysis (OR, 0.44; 95% CI, 0.36-0.54; p < 0.001) and multivariable analysis (OR, 0.37; 95% CI, 0.29-0.48; p < 0.001). The in-hospital mortality rate was 11.46% (204/1780) in the vitamin D group and 17.53% (1215/6930) in the no vitamin D group (p < 0.001). Vitamin D supplementation was associated with a lower in-hospital mortality rate in both univariable analysis (OR, 0.61; 95% CI, 0.52-0.71; p < 0.001) and multivariable analysis (OR, 0.57; 95% CI, 0.48-0.68; p < 0.001) (**Table 2**).

**Table 2:** The association of vitamin D supplementation with outcomes in the matched cohort.

Outcomes	Vitamin D (n=1780)	No vitamin D (n=6930)	Univariable analysis		Multivariable analysis^*^	
			HR/OR/MD(95%CI)	p-value	HR/OR/MD(95%CI)	p-value
Primary outcome						
28-day all-cause mortality^@^, n (%)	250 (14.0)	1546 (22.3)	0.59 (0.52-0.68)	<0.001	0.56 (0.49-0.64)	<0.001
Secondary outcomes						
ICU mortality^$^, n (%)	102 (5.7)	841 (12.1)	0.44 (0.36-0.54)	<0.001	0.37 (0.29-0.48)	<0.001
In-hospital mortality^$^, n (%)	204 (11.5)	1215 (17.5)	0.61 (0.52-0.71)	<0.001	0.57 (0.48-0.68)	<0.001
Length of ICU stay^¶^ (days), median [IQR]	3 [2, 6]	3 [2, 6]	0 (0-0)	0.90		
Length of hospital stay^¶^ (days), median [IQR]	10 [6, 18]	9 [5, 15]	1 (1-2)	<0.001		
Ventilation Duration^¶^ (days), median [IQR]	37 [14, 108]	39 [15, 109]	-1 (-3-2)	0.50		
CRRT Duration^¶^ (days), median [IQR]	80 [34, 184]	72 [19, 149]	8 (-3-28)	0.22		

CI, confidence interval; HR, hazard ratio; IQR, interquartile range; MD, median difference; OR, odds ratio; CRRT, continuous renal replacement therapy. ^*^Adjusted for age, race, BMI, APS-III, CCI, LODS, OASIS, GCS, MBP, respiratory rate, heart rate, temperature, hemoglobin, WBC, creatinine, AST, total bilirubin, pH, base excess, anion gap, INR. ^@^HR with 95% CI was calculated using Cox proportional hazards model. ^$^OR with 95% CI was calculated using logistic regression model. ^¶^MD with 95% CI was calculated using Hodges-Lehmann estimator.

#### Duration of MV and CRRT

The median duration of MV was 37 hours (IQR, 14-108) in the vitamin D group and 39 hours (IQR, 15-109) in the no vitamin D group. The median duration of CRRT was 80 hours (IQR, 34-184) in the vitamin D group and 72 hours (IQR, 19-149) in the no vitamin D group. No significant difference was observed between the vitamin D group and the no vitamin D group in the duration of MV (MD, -0.8 hours; 95% CI, -3.1-1.6; p = 0.50) and the duration of CRRT (MD, 8.0 hours; 95% CI, -3.1-28; p = 0.22) (**Table 2**).

#### Length of ICU stay and hospital stay

The median length of ICU stay was 3 days (IQR, 2-6) in the vitamin D group and 3 days (IQR, 2-6) in the no vitamin D group. The median length of hospital stay was 10 days (IQR, 6-18) in the vitamin D group and 9 days (IQR, 5-15) in the no vitamin D group. Vitamin D supplementation was not associated with a shortened length of ICU stay (MD, -0.01 days; 95% CI, -0.10-0.09; p = 0.90) but was associated with a prolonged length of hospital stay (MD, 1.2 days; 95% CI, 0.9-1.5; p < 0.001) (**Table 2**).

## Discussion

To date, there is no consensus on whether critically ill patients with sepsis would benefit from vitamin D supplementation to improve their prognosis. Therefore, we conducted a large retrospective cohort study using the MIMIC-IV database. The results of our study indicated that vitamin D supplementation may be associated with reduced 28-day all-cause mortality in critically ill patients with sepsis. Our findings were consistent in subgroup analyses and stable in sensitivity analyses, indicating the robustness of the results. Vitamin D supplementation was also associated with a significant reduction in ICU mortality and in-hospital mortality in both univariate and multivariate analyses. The prolonged ICU and hospital stay observed in the vitamin D group may be due to a competing relationship between ICU or in-hospital mortality and ICU or hospital stay. In conclusion, our study suggests that vitamin D supplementation during the ICU stay may improve the prognosis of patients with sepsis.

### Relation with previous evidence

In recent years, the relationship between vitamin D and all-cause mortality has become a spotlight topic of growing research interest. Vitamin D deficiency in critically ill hospitalized patients is considered to be a risk factor for sepsis and is associated with higher mortality rates (Braun et al., 2011; Malinverni et al., 2023; Seok et al., 2023; Vanichkulbodee et al., 2023). Specifically, Moromizato et al. reported that patients with pre-admission 25-hydroxyvitamin D levels below 15 ng/mL were more susceptible to sepsis and experienced higher 90-day mortality (Moromizato et al., 2014). In 2018, Zhou and colleagues conducted a meta-analysis of 24 studies to investigate the association between serum vitamin D levels and sepsis (Zhou et al., 2018). They found that patients with sepsis exhibited lower vitamin D levels than those without sepsis, but vitamin D deficiency was not significantly associated with increased sepsis-related mortality (Zhou et al., 2018). However, a recent systematic review of 8 studies showed that lower 25-hydroxyvitamin D levels were independently related to greater mortality in septic patients (Li and Ding, 2020). Subsequent subgroup analyses found that only severe vitamin D deficiency (<10 ng/ml) was associated with higher mortality in sepsis, whereas no such association was observed in individuals with higher 25-hydroxyvitamin D levels (Li and Ding, 2020). Further research is therefore needed to determine whether vitamin D deficiency is involved in the pathogenesis of infection and sepsis, or whether vitamin D status can be used as a marker of severity in patients with sepsis.

### Possible explanations for findings

Although further research is needed to better understand the specific mechanisms, several possible explanations for the beneficial effects of vitamin D supplementation on sepsis have been proposed. The beneficial effects of vitamin D on infectious diseases may be attributed to its potential immunomodulatory properties (Murdaca and Gangemi, 2022; Miao and Goltzman, 2023). First, in innate immunity, by binding to its receptor, vitamin D promotes the transcription of the antibacterial peptide gene and increases the expression of LL-37 (Liu et al., 2006). In addition, vitamin D suppresses the expression of Toll-like receptor (TLR) on the surface of monocytes, thereby preventing an exaggerated immune response to pathogen-associated molecular patterns and contributing to the alleviation of sepsis (Sadeghi et al., 2006). Second, in terms of adaptive immunity, researchers have found that intravenous usage of calcitriol after sepsis could modulate the homeostasis of CD4+ T-cells and reduce sepsis-induced kidney injury in obese mice (Yeh et al., 2022). Vitamin D also inhibits pokeweed mitogen-stimulated human B-cell activation (Shiozawa et al., 1985). Therefore, vitamin D plays a crucial role in maintaining immune homeostasis and protecting organs from damage caused by excessive immune responses in sepsis. Third, vitamin D can suppress systemic inflammation and enhance antioxidant capacity in patients with sepsis. Reports suggest that vitamin D can ameliorate endotoxemia and improve the prognosis in lipopolysaccharide (LPS) treated mice by regulating free radicals and thromboxane A2 (TXA2) formation (Horiuchi et al., 1991). Furthermore, vitamin D has been demonstrated to protect mice against oxidative damage by activating the Nrf2-HO-1 signaling pathway and inhibiting the phosphorylation of NF-kappaB (Zhang et al., 2021). Notably, the association between high-dose vitamin D supplementation and improved clinical outcomes in patients with COVID-19 has been established through the suppression of hyperinflammatory cytokine storms (Sarhan et al., 2022).

### Implications for clinical practice

For many years, vitamin D has been recommended to maintain calcium balance and enhance musculoskeletal health. Recently, beyond its importance in endocrine function, a growing body of evidence showed that vitamin D plays an important role in critical care settings. According to a comprehensive review, vitamin D may lower all-cause mortality in individuals with COVID-19 or liver disease, as well as cause-specific mortality in patients with respiratory cancer (Cao et al., 2023). Vitamin D plays a key role in immunomodulation, particularly in the context of autoimmunity (Triantos et al., 2022), by attenuating the inflammatory response, inducing phagocytosis, and promoting lymphocyte proliferation (Vanichkulbodee et al., 2023). Animal studies also showed that vitamin D might mitigate sepsis-induced acute lung injury by inhibiting endoplasmic reticulum stress (Ahmad et al., 2022). Therefore, vitamin D supplementation is a promising adjunctive therapy for sepsis in clinical settings.

Several observational studies have shown that vitamin D deficiency is common in patients admitted to the ICU, with reported rates ranging from 40% to 70% (Amrein et al., 2018). Despite the high prevalence of vitamin D deficiency in critically ill patients, routine vitamin D supplementation strategy is not practiced in ICU. Our study showed that vitamin D could improve clinical outcomes, highlighting the need for closer monitoring and proactive management of vitamin D in critically ill patients, especially those with sepsis. As the current study is based on a retrospective database, the cause-and-effect relationship is uncertain. Patients with both vitamin D sufficiency and insufficiency were included in the current study, but we believe that future studies should analyze these subpopulations separately. To better clarify the role of vitamin D supplementation in different critically ill patient populations, further validation studies are also needed. A paradoxical finding in this study is that vitamin D supplementation is associated with reduced mortality but a prolonged hospital stay. Such a discrepancy may be due to the competition between mortality and hospital stay.

### Study limitations

This study has some limitations. First, due to the retrospective observational design of our study, the results are susceptible to residual bias and unmeasured confounders despite the use of rigorous methodologies such as PSM and multivariable analyses. Second, the study covers a long period, during which substantial improvements have occurred in sepsis management, thus exposing our study to bias. Third, due to the missing data in the MIMIC-IV database, we were not able to obtain 25-hydroxyvitamin D concentration measurements at different time points for all participants, and the relationship between 25-hydroxyvitamin D levels, supplementation practices, and patient outcomes was not thoroughly evaluated. Fourth, the indications for initiation and discontinuation of vitamin D were not available in the MIMIC-IV database, so we did not evaluate the effect of timing, dose and route of vitamin D administration on the prognosis of patients with sepsis. Finally, we did not evaluate the safety of vitamin D because adverse effects of vitamin D supplementation outside the ICU are very rare.

## Conclusions

In summary, vitamin D supplementation was associated with lower 28-day all-cause mortality, in-hospital mortality and ICU mortality in critically ill patients with sepsis. Prospective studies are needed to verify this retrospective finding.

## Data Availability

Publicly available datasets were analyzed in this study. This data can be found here: https://physionet.org/content/mimiciv/2.0/.

## Supplementary material

Supplementary Figure 1 Percentage of missing data of each variable.

Supplementary Figure 2 Variance inflation factor of each variable in the matched cohort.

Supplementary Figure 3 Variance inflation factor of each variable in the unmatched cohort.

Supplementary Figure 4 Association Between serum 25-hydroxyvitamin D and 28-day mortality Using a Restricted Cubic Spline Regression Model. Graphs show HRs for 28-day mortality according to serum 25-hydroxyvitamin D. Data were fitted by a restricted cubic spline Cox proportional hazards regression model, and the model was conducted with 4 knots at the 5th, 35th, 65th, 95th percentiles of serum 25-hydroxyvitamin D (reference is the median). Solid lines indicate HRs, and shadow shape indicate 95% CIs. HR, hazard ratio; CI, confidence interval.

Supplementary Table 4 Cox regression model for 28-day all-cause mortality using stepwise selection in the matched cohort.

Supplementary Table 5 Cox regression model for 28-day all-cause mortality using stepwise selection in the unmatched cohort.

## Glossary

MIMIC-IV: Medical Information Mart for Intensive Care IV
ICU: Intensive Care Unit
PSM: Propensity Score Matching
HR: Hazard Ratio
CI: Confidence Interval
OR: Odds Ratio
RCTs: Randomized Controlled Trials
MV: Mechanical Ventilation
STROBE: Strengthening the Reporting of Observational Studies in Epidemiology
IV: IntraVeneous administration
PO: Oral administration
NG: NasoGastric feeding
CRRT: Continuous Renal Replacement Therapy
SQL: Structured Query Language
BMI: Body Mass Index
RR: Respiratory Rate
MBP: Mean Blood Pressure
WBC: White Blood Cell
BUN: Blood Urea Nitrogen
ALT: Alanine Aminotransferase
AST: Aspartate Aminotransferase
pH: Potential of Hydrogen
pO2: partial pressure of Oxygen
pCO2: partial pressure of Carbon Dioxide
INR: International Normalized Ratio
SD: Standard Deviation
IQR: Interquartile Range
VIF: Variance Inflation Factor
MD: Median Difference
GCS: Glasgow Coma Scale
SMD: Standardized Mean Difference
APS-III: Acute Physiology Score III
LODS: Logistic Organ Dysfunction System
OASIS: Oxford Acute Severity of Illness Score
SOFA: Sequential Organ Failure Assessment Score
OI: Oxygenation Index
PaO2/FiO2 ratio: the partial pressure of arterial oxygen to fraction of inspired oxygen ratio
CCI: Charlson Comorbidity Index
TXA2: Thromboxane A2
LPS: Lipopolysaccharide
TLR: Toll-like receptor
