# Supplementary material for: Vitamin D supplementation during intensive care unit stay is associated with improved outcomes in critically Ill patients with sepsis: a cohort study
Source: Front Cell Infect Microbiol. 2025 Jan 20;14:1485554. doi: 10.3389/fcimb.2024.1485554 (PMC11788162; doi:10.3389/fcimb.2024.1485554)
Supplement: Supplementary file 1 [file DataSheet1.pdf]

Dependent: Surv(Time, Status)		all	HR (univariable)	HR (multivariable)	HR (final)
Age	<=60	2046 (23.5%)			
	>60	6664 (76.5%)	1.82 (1.60-2.07, p<.001)	1.20 (1.03-1.39, p=.020)	1.19 (1.03-1.39, p=.021)
Gender	F	4313 (49.5%)			
	M	4397 (50.5%)	1.02 (0.93-1.12, p=.642)		
Race	BLACK	904 (10.4%)			
	OTHER	820 (9.4%)	0.99 (0.79-1.24, p=.933)	0.98 (0.78-1.22, p=.828)	0.98 (0.79-1.23, p=.875)
	UNKNOWN	823 (9.4%)	1.89 (1.56-2.30, p<.001)	1.66 (1.36-2.02, p<.001)	1.66 (1.36-2.02, p<.001)
	WHITE	6163 (70.8%)	1.09 (0.92-1.28, p=.320)	1.12 (0.95-1.32, p=.180)	1.11 (0.94-1.31, p=.200)
BMI	Mean ± SD	28.6 ± 6.1	0.98 (0.98-0.99, p<.001)	0.98 (0.97-0.99, p<.001)	0.98 (0.97-0.99, p<.001)
APS.III	Mean ± SD	51.4 ± 20.7	1.03 (1.03-1.03, p<.001)	1.01 (1.01-1.02, p<.001)	1.01 (1.01-1.02, p<.001)
Charlson.Comorbidity.Index	Mean ± SD	6.0 ± 2.9	1.19 (1.17-1.20, p<.001)	1.15 (1.13-1.17, p<.001)	1.15 (1.13-1.17, p<.001)
LODS	Mean ± SD	5.9 ± 3.3	1.23 (1.22-1.25, p<.001)	1.13 (1.11-1.15, p<.001)	1.13 (1.11-1.16, p<.001)
OASIS	Mean ± SD	34.5 ± 9.0	1.07 (1.07-1.08, p<.001)	1.02 (1.01-1.02, p<.001)	1.01 (1.01-1.02, p<.001)
SOFA	Mean ± SD	6.1 ± 3.4	1.16 (1.15-1.18, p<.001)	1.01 (0.99-1.04, p=.393)	
GCS	Mean ± SD	13.5 ± 2.7	0.93 (0.92-0.95, p<.001)	1.05 (1.03-1.07, p<.001)	1.05 (1.03-1.07, p<.001)
MBP	Mean ± SD	75.9 ± 10.2	0.98 (0.97-0.98, p<.001)	0.99 (0.99-1.00, p=.001)	0.99 (0.99-1.00, p=.002)
Resp.Rate	Mean ± SD	19.8 ± 4.0	1.08 (1.07-1.09, p<.001)	1.04 (1.03-1.05, p<.001)	1.04 (1.03-1.05, p<.001)
Heart.Rate	Mean ± SD	86.2 ± 16.1	1.01 (1.01-1.02, p<.001)	1.01 (1.01-1.01, p<.001)	1.01 (1.01-1.01, p<.001)
Temperature	Mean ± SD	36.9 ± 0.6	0.63 (0.58-0.67, p<.001)	0.71 (0.65-0.76, p<.001)	0.71 (0.65-0.76, p<.001)
Hemoglobin	Mean ± SD	9.5 ± 2.1	0.95 (0.93-0.97, p<.001)	1.05 (1.03-1.08, p<.001)	1.05 (1.03-1.08, p<.001)
Platelets	Mean ± SD	181.0 ± 110.1	1.00 (1.00-1.00, p=.666)		
WBC	Mean ± SD	15.3 ± 13.5	1.01 (1.01-1.01, p<.001)	1.01 (1.00-1.01, p<.001)	1.01 (1.00-1.01, p<.001)
BUN	Mean ± SD	36.7 ± 27.8	1.01 (1.01-1.01, p<.001)	1.00 (1.00-1.00, p=.818)	
Creatinine	Mean ± SD	2.0 ± 2.1	1.06 (1.04-1.08, p<.001)	0.92 (0.88-0.95, p<.001)	0.92 (0.89-0.95, p<.001)
ALT	Mean ± SD	158.6 ± 845.6	1.00 (1.00-1.00, p=.146)		
AST	Mean ± SD	249.3 ± 1006.5	1.00 (1.00-1.00, p<.001)	1.00 (1.00-1.00, p=.078)	1.00 (1.00-1.00, p=.137)
Total.Bilirubin	Mean ± SD	2.3 ± 4.5	1.04 (1.03-1.04, p<.001)	1.02 (1.02-1.03, p<.001)	1.03 (1.02-1.03, p<.001)
Glucose	Mean ± SD	145.8 ± 56.4	1.00 (1.00-1.00, p<.001)	1.00 (1.00-1.00, p=.893)	
pH	Mean ± SD	7.3 ± 0.1	0.15 (0.09-0.25, p<.001)	1.49 (0.71-3.11, p=.293)	
pO2	Mean ± SD	109.6 ± 50.9	0.99 (0.99-1.00, p<.001)	1.00 (1.00-1.00, p=.852)	
pCO2	Mean ± SD	45.0 ± 11.9	1.00 (0.99-1.00, p=.179)		
PaO2.FiO2.Ratio	Mean ± SD	227.8 ± 91.7	1.00 (1.00-1.00, p<.001)	1.00 (1.00-1.00, p=.756)	
Base.Excess	Mean ± SD	-2.5 ± 4.7	0.95 (0.94-0.96, p<.001)	1.03 (1.02-1.05, p<.001)	1.04 (1.03-1.05, p<.001)
Lactate	Mean ± SD	2.5 ± 1.9	1.15 (1.13-1.17, p<.001)	1.02 (0.99-1.05, p=.197)	
Calcium	Mean ± SD	8.0 ± 0.9	0.99 (0.94-1.04, p=.699)		
Sodium	Mean ± SD	136.3 ± 5.9	1.00 (0.99-1.01, p=.673)		
Potassium	Mean ± SD	4.7 ± 0.9	1.21 (1.16-1.27, p<.001)	1.01 (0.96-1.06, p=.742)	
Chloride	Mean ± SD	101.2 ± 7.3	0.98 (0.97-0.99, p<.001)	1.00 (0.99-1.01, p=.931)	
Anion.Gap	Mean ± SD	17.2 ± 5.2	1.07 (1.06-1.08, p<.001)	1.03 (1.02-1.04, p<.001)	1.03 (1.02-1.04, p<.001)
INR	Mean ± SD	1.7 ± 1.3	1.13 (1.11-1.15, p<.001)	1.02 (0.99-1.05, p=.130)	1.02 (0.99-1.05, p=.128)
Antibiotic.Lag	Mean ± SD	14.8 ± 17.7	1.00 (1.00-1.01, p=.005)	1.00 (1.00-1.00, p=.239)	
First.Day.Vasopressor	No	6176 (70.9%)			
	Yes	2534 (29.1%)	1.82 (1.65-2.00, p<.001)	0.94 (0.82-1.07, p=.356)	
VitaminD	No	6930 (79.6%)			
	Yes	1780 (20.4%)	0.59 (0.52-0.68, p<.001)	0.56 (0.49-0.64, p<.001)	0.56 (0.49-0.65, p<.001)

n=8710, events=1796, Likelihood ratio test=2076.93 on 34 df(p<.001)
cluster=subclass
