# Supplementary figures and images for: Vitamin D supplementation during intensive care unit stay is associated with improved outcomes in critically Ill patients with sepsis: a cohort study
Source: Front Cell Infect Microbiol. 2025 Jan 20;14:1485554. doi: 10.3389/fcimb.2024.1485554 (PMC11788162; doi:10.3389/fcimb.2024.1485554)

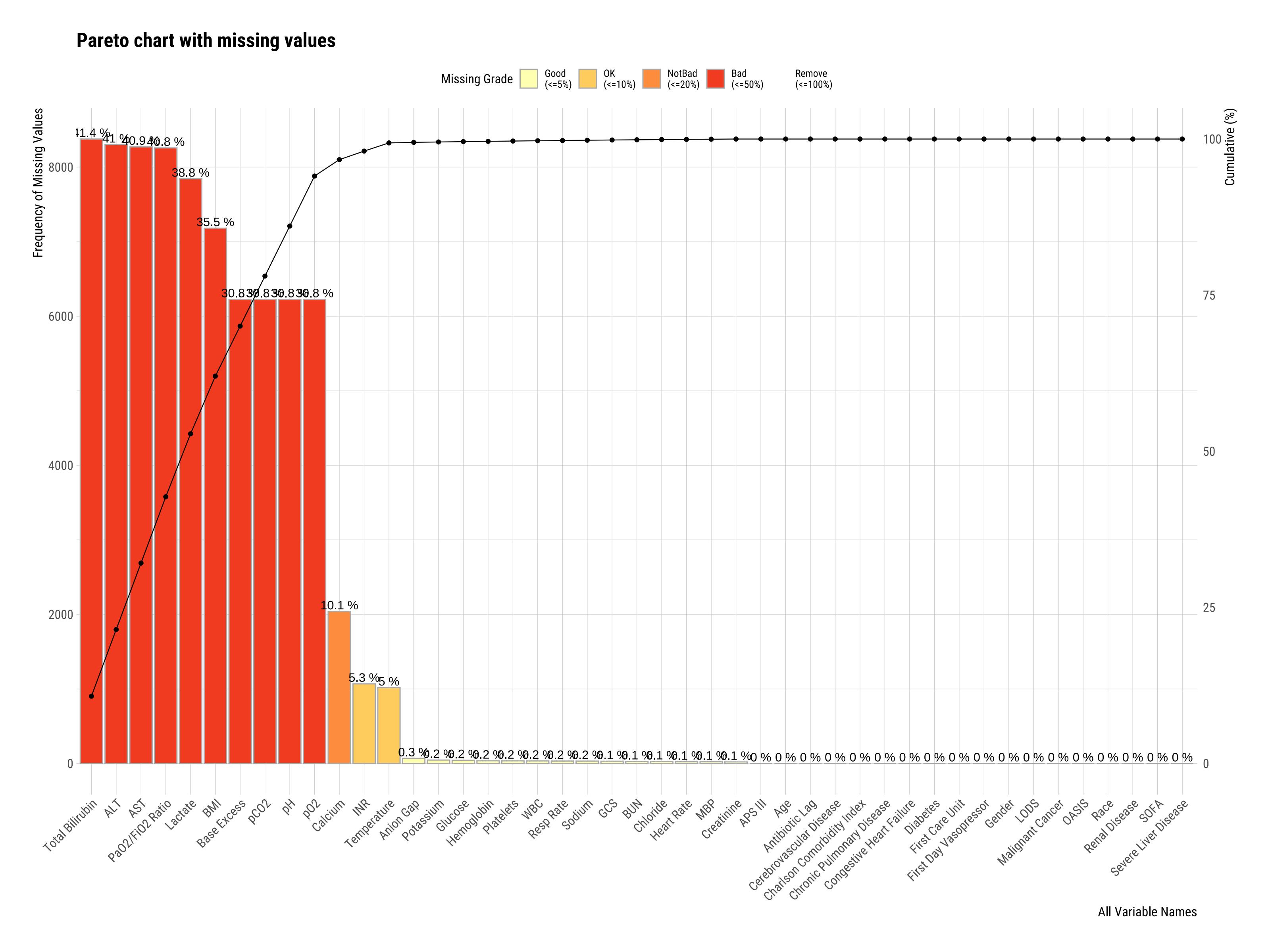

Supplement: Supplementary Figure 1 — Percentage of missing data of each variable. [file Image1.jpeg]

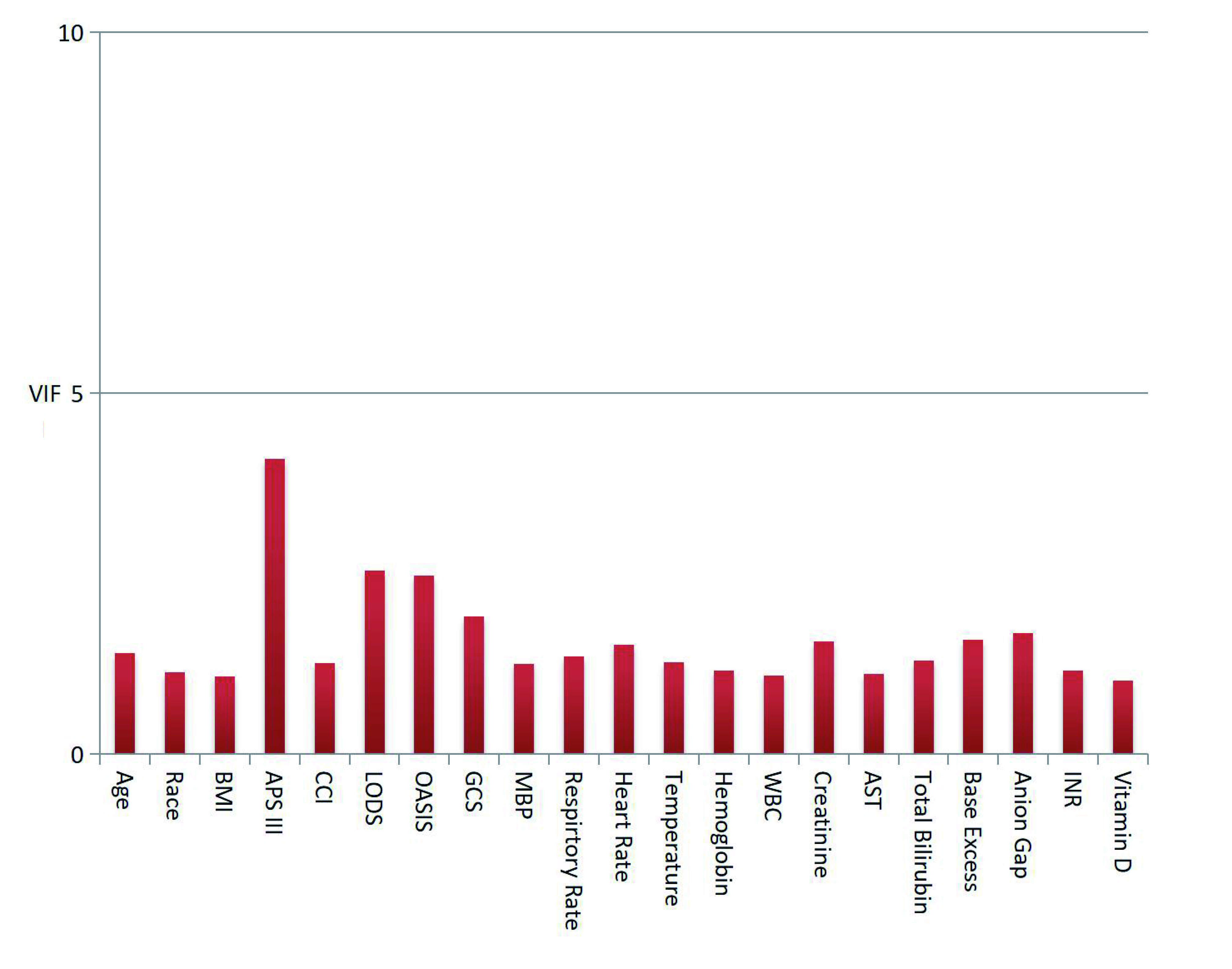

Supplement: Supplementary Figure 2 — Variance inflation factor of each variable in the matched cohort. [file Image2.jpeg]

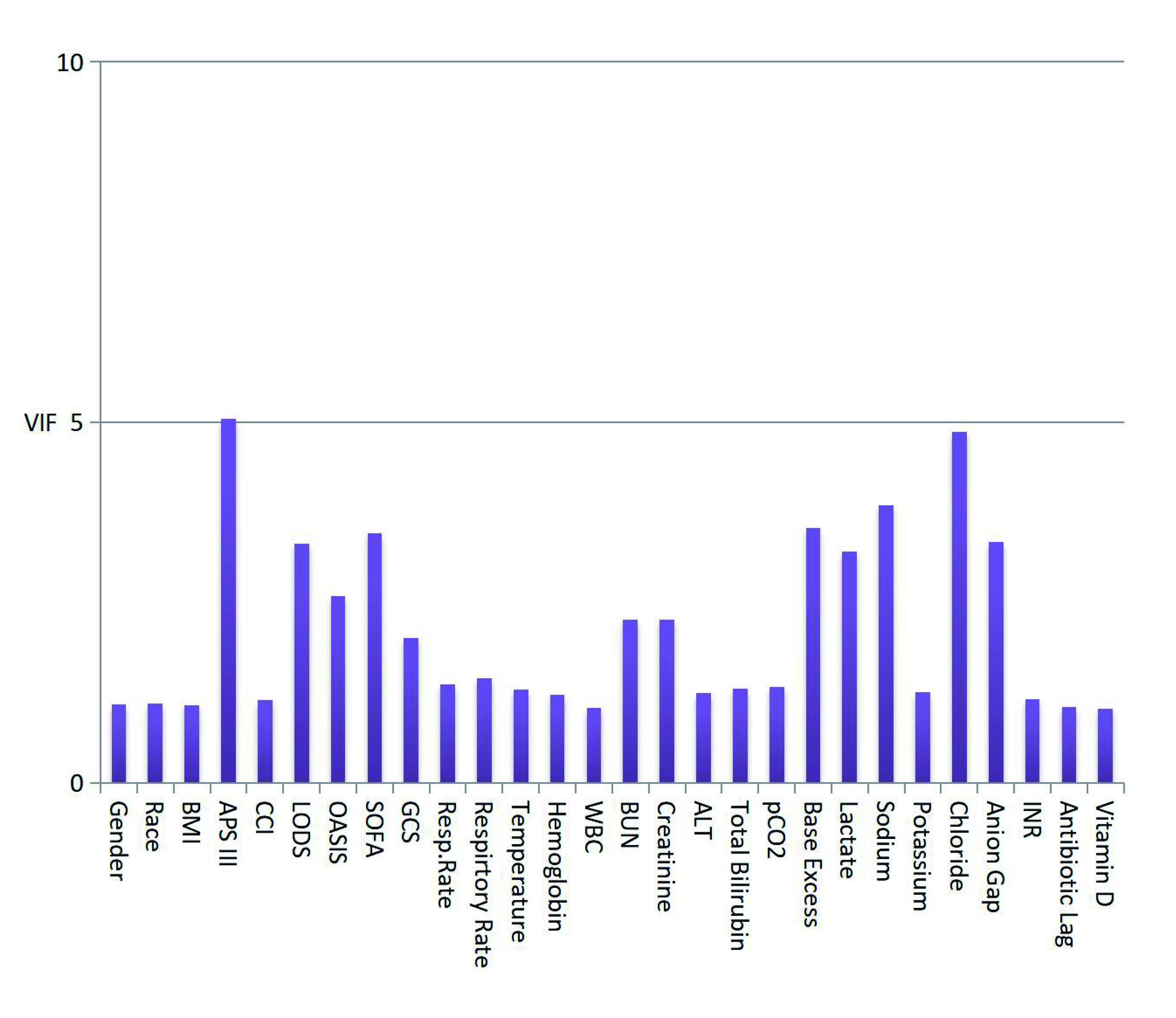

Supplement: Supplementary Figure 3 — Variance inflation factor of each variable in the unmatched cohort. [file Image3.jpeg]

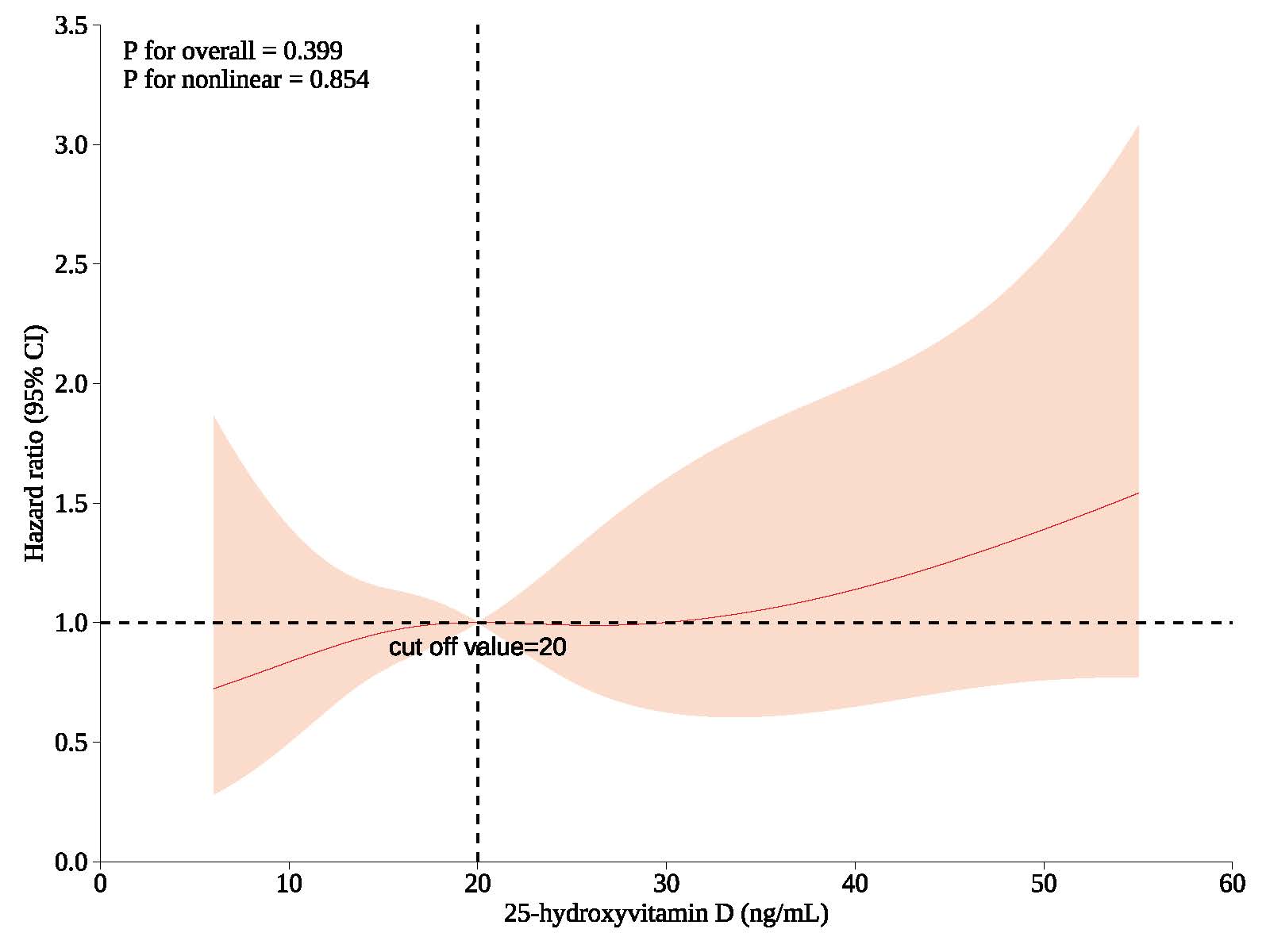

Supplement: Supplementary Figure 4 — Association Between serum 25-hydroxyvitamin D and 28-day mortality Using a Restricted Cubic Spline Regression Model. Graphs show HRs for 28-day mortality according to serum 25-hydroxyvitamin D. Data were fitted by a restricted cubic spline Cox proportional hazards regression model, and the model was conducted with 4 knots at the 5th, 35th, 65th, 95th percentiles of serum 25-hydroxyvitamin D (reference is the median). Solid lines indicate HRs, and shadow shape indicate 95% CIs. HR, hazard ratio; CI, confidence interval. [file Image4.jpeg]
