# Supplementary material for: Vitamin D supplementation during intensive care unit stay is associated with improved outcomes in critically Ill patients with sepsis: a cohort study
Source: Front Cell Infect Microbiol. 2025 Jan 20;14:1485554. doi: 10.3389/fcimb.2024.1485554 (PMC11788162; doi:10.3389/fcimb.2024.1485554)
Supplement: Supplementary Table 5 — Cox regression model for 28-day all-cause mortality using stepwise selection in the unmatched cohort. [file Table2.docx]

Table S2. Variance inflation factor of each variable in the matched cohort.

| Variables | Variance inflation factor(VIF) |
| --- | --- |
| Age | 1.40 |
| Race | 1.13 |
| BMI | 1.07 |
| APS III | 4.10 |
| CCI | 1.26 |
| LODS | 2.55 |
| OASIS | 2.48 |
| GCS | 1.91 |
| MBP | 1.25 |
| Respiratory Rate | 1.36 |
| Heart Rate | 1.51 |
| Temperature | 1.28 |
| Hemoglobin | 1.16 |
| WBC | 1.09 |
| Creatinine | 1.56 |
| AST | 1.11 |
| Total Bilirubin | 1.30 |
| Base Excess | 1.58 |
| Anion Gap | 1.67 |
| INR | 1.16 |
| Vitamin D | 1.03 |
