# Supplementary material for: Vitamin D supplementation during intensive care unit stay is associated with improved outcomes in critically Ill patients with sepsis: a cohort study
Source: Front Cell Infect Microbiol. 2025 Jan 20;14:1485554. doi: 10.3389/fcimb.2024.1485554 (PMC11788162; doi:10.3389/fcimb.2024.1485554)
Supplement: Supplementary file 9 [file Table3.docx]

Table S3. Variance inflation factor of each variable in the unmatched cohort.

| Variables | Variance inflation factor (VIF) |
| --- | --- |
| Gender | 1.09 |
| Race | 1.10 |
| BMI | 1.08 |
| APS III | 5.05 |
| CCI | 1.16 |
| LODS | 3.32 |
| OASIS | 2.59 |
| SOFA | 3.46 |
| GCS | 2.01 |
| Respiratory Rate | 1.37 |
| Respirtory Rate | 1.45 |
| Temperature | 1.29 |
| Hemoglobin | 1.22 |
| WBC | 1.04 |
| BUN | 2.26 |
| Creatinine | 2.26 |
| ALT | 1.25 |
| Total Bilirubin | 1.31 |
| pCO2 | 1.33 |
| Base Excess | 3.53 |
| Lactate | 3.21 |
| Sodium | 3.85 |
| Potassium | 1.26 |
| Chloride | 4.86 |
| Anion Gap | 3.34 |
| INR | 1.17 |
| Antibiotic Lag | 1.05 |
| Vitamin D | 1.03 |
